# ﻿Weight as a driver of ontogenetic color pattern shift in the King Ratsnake *Elaphe carinata carinata* (Günther, 1864) (Reptilia, Serpentes, Colubridae)

**DOI:** 10.3897/zookeys.1253.143797

**Published:** 2025-09-23

**Authors:** Zhihao Jiang, Yi Zhang, Wei Zhao, Song Huang, Ruyi Huang, Tierui Zhang, Jing Yu, Yanan Gong, Zhangbo Cui, Zicheng Su, Xinge Wang, Jinmin Chen

**Affiliations:** 1 The Anhui Provincial Key Laboratory of Biodiversity Conservation and Ecological Security in the Yangtze River Basin, College of Life Sciences, Anhui Normal University, Wuhu 241000, China Anhui Normal University Wuhu China; 2 Shanghai Collaborative Innovation for Aquatic Animal Genetics and Breeding, Shanghai Ocean University, Shanghai 201306, China Shanghai Ocean University Shanghai China; 3 Shanghai Wildlife and Protected Natural Areas Research Center, Shanghai 200336, China Shanghai Wildlife and Protected Natural Areas Research Center Shanghai China; 4 School of Life Sciences, Nanjing Forestry University, Nanjing 210037, Jiangsu, China Nanjing Forestry University Nanjing China

**Keywords:** Captive breeding, full-pattern, morphology, ontogenetic, semi-pattern

## Abstract

In many snakes, the color pattern of neonates differs from that of adults, often superficially resembling a separate species. This phenomenon is known as ontogenetic color pattern shift. Ontogenetic shift may be associated with changes in body weight or age. We addressed this question using the King Ratsnake, *Elaphe carinata carinata* (Günther, 1864), a species that shows ontogenetic changes in color pattern. Five gravid King Ratsnakes were collected in June 2021 at Huangshan City, Anhui Province, China. Sixty-six fertilized eggs were laid in July, and all eggs hatched and produced neonates in September 2021. Both weight and age since birth were recorded before every feeding, over a period of 22 months. We observed marked ontogenetic color change as snakes grew. As the body weight of the neonates reached ~40 g, the color pattern gradually became a “semi-pattern”, where only the color pattern on the front half of the body resembled that of adults. When the body weight reached ~80 g, the “semi-pattern” gradually became a “full-pattern”, where the color pattern over the whole body was consistent with that of adults. A subset of individuals did not take food, and thus did not grow at all. These individuals experienced no ontogenetic color change, though they were the same age as ones who did, but were much smaller. Our results show that the color pattern shift in King Ratsnakes is likely associated with body weight, but not age.

## ﻿Introduction

For many years, the diversity of snake coloration has attracted the attention of biologists (Booth 1990; Mouy 2024). Many studies have addressed topics such as sexual dimorphisms (e.g., Ji et al. 1997; King et al. 1999; Vincent et al. 2004; Hendry et al. 2014; Borczyk et al. 2024; Pokorný et al. 2024) and intraspecific color pattern polymorphisms (van Rooijen et al. 2011; Kuriyama et al. 2020; Farooq and Uetz 2021), but few (e.g., Myers 1962; Cox and Davis Rabosky 2023) have studied factors related to ontogenetic color pattern shift in snakes. Ontogenetic color pattern shift, a common phenomenon in some snakes, is defined as: the color pattern of a neonate is radically different from its adult form, superficially resembling a different species.

The King Ratsnake belongs to the family Colubridae, which has three subspecies: *Elaphe carinata carinata* (Günther, 1864), *E. c. yonaguniensis* Takara, 1962, and *E. c. deqinensis* Yang & Su, 1984. The subspecies *E. c. carinata* is mainly distributed in China, as far south as Guangdong, Guangxi and Taiwan Province, to as far north as Beijing, Shanxi and Gansu Province (Huang 2021; Uetz et al. 2024). *Elaphe c. carinata* mainly inhabits mountainous and hilly areas, and generally feeds on rodents, birds, and eggs. Here we used the King Ratsnake, *E. c. carinata* to examine the process of color pattern shifts, and explore whether this is related to weight or growth time. *Elaphe c. carinata* is an excellent model organism to examine color pattern shifts as it has a variable color pattern showing three specific life stages (neonatal-pattern, semi-pattern and full-pattern).

## ﻿Material and methods

### ﻿Sampling

Five gravid individuals of *E. c. carinata* (sampling numbers: HSR21061–65) were collected in June 2021 at Huangshan City (29.4°–30.03°N, 117.65°–118.43°E; elevation 145–523 m), Anhui Province, China and brought back to the lab at Anhui Normal University.

Collections of all animals used in this study obey the Wildlife Protection Act of China, following the guidelines and regulations approved by the internal review board of AHNU, and with the permission of local government authorities.

### ﻿Feeding Trials

Gravid snakes were kept separately in transparent plastic containers (120×80×50 cm) with air-ventilation holes. China fir wood chips were used as a substrate for the cages with a depth of 3–4 cm. A wooden hiding box (30×25×25 cm) and water box (20×15×8 cm) were provided for the snakes to rest and obtain water. The cages were misted with water daily to maintain a humidity of 85%. The room temperature was controlled to ~28 °C, and gradually reduced to 12 °C in December for hibernation and increased to 28 °C in March of the next year. The duration of light, 13 h from June to December and 11 h in the remaining months, was like the seasonal conditions in the natural habitat (Parmar and Limbachiya 2020). Three euthanized mice (each mouse 20–25 g) were offered to each snake every seven days.

**Figure 1.**
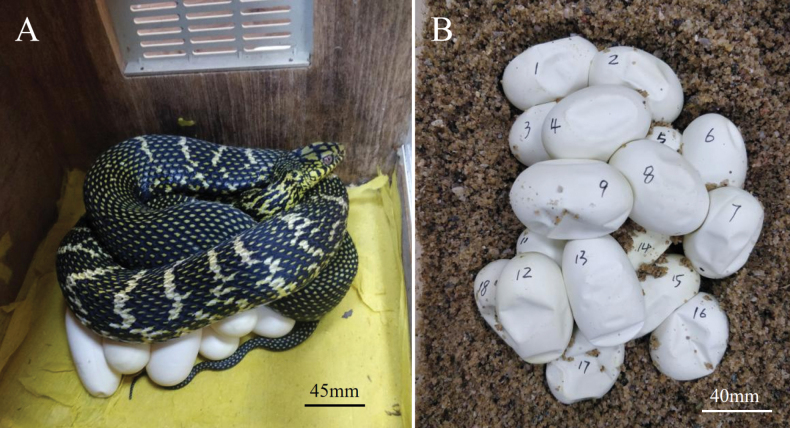
A. Female *Elaphe c. carinata* (HSR21061) after laying eggs on the substrate of biaoxin paper in egg-laying container; B. Eggs (laid by HSR21064) and the substrate of wet vermiculite in the incubation container.

When we observed that gravid snakes rejected a mouse, it was moved to an egg-laying container, a special wooden box (50×28×30 cm). We used sterile biaoxin paper as a substrate to prevent dehydration or bacterial/fungal infection of eggs (Fig. 1A). After the end of egg-laying, we marked the upper surfaces with an alcohol-free marker to avoid rotating the eggs during transfer and handling (Zohari et al. 2024). The bottom of a plastic incubation box (28×18×15 cm) was covered with ~10 cm of wet vermiculite, which when dry, had been previously mixed with cooled, boiled water at a ratio of about 1:1 by weight (Fig. 1B).

Upon parturition, neonatal King Ratsnakes were transferred to new plastic boxes (28×18×15 cm) after hatching, with the specific date of hatch and initial weights recorded. We used urine pads (33×45 cm) as substrate to keep the box clean, and provided suitable water boxes and shelter boxes for them to rest and drink. Additionally, rough stones were placed in these boxes to aid shedding. The snakes were fed mice (about 10% of snake’s weight with assisted feeding) every 7–10 days. Both weight and dates were recorded before every feeding. Mice that were not eaten would be removed after 12 h.

### ﻿Statistical analysis

Statistical analyses were carried out using IBM SPSS Statistics 27.0 (Junaidi 2015). Relationships between three specific life stages and the weight of the King Ratsnake were visualized using boxplots (Fig. 3). The weight data were divided into three groups based on the color pattern: neonatal-pattern (NP) refers to the first color pattern in life; semi-pattern (SP) refers to the second color pattern, and full-pattern (FP) refers to the last color pattern observed throughout all life stages.

## ﻿Results

Five *E. c. carinata* laid a total of 66 eggs from 6 July to 23 July 2021. From 26 August to 13 September 2021, 66 neonatal King Ratsnakes were hatched. The range of neonatal mass was 19.53–26.62 g, and average mass was 23.09 g on September 15, 2021. The first sheds after hatching of the neonatal snakes were ~7–10 days later. Weight data are listed in Table 1; 34.8% individuals died before adulthood.

**Table 1.** The weight data (g) of the King Ratsnake on specific dates were measured with a digital balance to the nearest 0.1g. “D” indicates that the snake died. Letter in “()” means the three different specific life stages, “N” means neonatal-pattern, “S” means semi-pattern and “F” means full-pattern.

Numbers	Voucher	2021.9.15	2021.12.29	2022.3.29	2022.5.17	2022.6.17	2022.8.29	2023.3.24
1	HSR21061-1	22.44 (N)	14.20 (N)	D	-	-	-	-
2	HSR21061-2	23.33 (N)	25.56 (N)	19.12 (N)	29.72 (N)	44.53 (S)	35.89 (S)	42.32 (S)
3	HSR21061-3	26.45 (N)	22.17 (N)	18.91 (N)	23.75 (N)	41.67 (S)	51.62 (S)	65.22 (S)
4	HSR21061-4	26.14 (N)	20.52 (N)	13.33 (N)	D	-	-	-
5	HSR21061-5	22.47 (N)	22.93 (N)	18.70 (N)	21.51 (N)	37.32 (S)	51.54 (S)	68.89 (S)
6	HSR21061-6	23.64 (N)	13.12 (N)	10.23 (N)	D	-	-	-
7	HSR21061-7	26.53 (N)	20.41 (N)	17.90 (N)	28.13 (N)	41.73 (S)	47.80 (S)	56.71 (S)
8	HSR21061-8	22.08 (N)	25.50 (N)	21.91 (N)	25.41 (N)	44.53 (S)	54.28 (S)	59.33 (S)
9	HSR21061-9	26.23 (N)	26.72 (N)	24.44 (N)	30.91 (N)	66.50 (S)	77.01 (F)	90.35 (F)
10	HSR21061-10	23.31 (N)	28.60 (N)	26.15 (N)	38.33 (S)	53.80 (S)	91.65 (F)	113.28 (F)
11	HSR21062-1	20.11 (N)	19.10 (N)	13.12 (N)	D	-	-	-
12	HSR21062-2	25.23 (N)	21.22 (N)	17.20 (N)	20.71 (N)	24.91 (N)	25.13 (N)	24.77 (N)
13	HSR21062-3	22.36 (N)	19.56 (N)	12.92 (N)	D	-	-	-
14	HSR21062-4	22.69 (N)	19.90 (N)	12.30 (N)	D	-	-	-
15	HSR21062-5	25.33 (N)	20.08 (N)	18.01 (N)	24.33 (N)	39.47 (S)	52.20 (S)	70.28 (F)
16	HSR21062-6	24.34 (N)	21.13 (N)	17.34 (N)	20.25 (N)	33.56 (S)	48.97 (S)	66.53 (S)
17	HSR21062-7	25.55 (N)	18.23 (N)	15.80 (N)	11.93 (N)	D	-	-
18	HSR21062-8	24.85 (N)	21.22 (N)	15.41 (N)	12.02 (N)	D	-	-
19	HSR21062-9	26.41 (N)	23.54 (N)	18.50 (N)	22.60 (N)	26.21 (N)	23.24 (N)	27.55 (N)
20	HSR21063-1	22.76 (N)	16.20 (N)	12.33 (N)	D	-	-	-
21	HSR21063-2	22.45 (N)	17.15 (N)	12.50 (N)	D	-	-	-
22	HSR21063-3	20.07 (N)	17.90 (N)	17.12 (N)	20.10 (N)	23.05 (N)	19.51 (N)	22.34 (N)
23	HSR21063-4	23.96 (N)	17.61 (N)	20.53 (N)	20.63 (N)	26.12 (N)	40.03 (S)	53.68 (S)
24	HSR21063-5	19.53 (N)	15.92 (N)	17.80 (N)	25.71 (N)	45.05 (S)	52.25 (S)	70.12 (S)
25	HSR21063-6	20.13 (N)	16.21 (N)	15.53 (N)	18.07 (N)	55.71 (S)	93.43 (F)	121.19 (F)
26	HSR21063-7	20.89 (N)	17.23 (N)	16.51 (N)	22.12 (N)	30.13 (N)	28.62 (N)	30.26 (N)
27	HSR21063-8	23.58 (N)	19.64 (N)	15.63 (N)	29.30 (N)	72.61 (S)	102.03 (F)	133.94 (F)
28	HSR21063-9	24.15 (N)	22.17 (N)	15.31 (N)	31.92 (N)	62.03 (S)	109.37 (F)	137.81 (F)
29	HSR21063-10	20.78 (N)	18.33 (N)	14.20 (N)	21.22 (N)	31.83 (N)	24.05 (N)	28.33 (N)
30	HSR21063-11	21.67 (N)	20.91 (N)	18.02 (N)	22.23 (N)	55.62 (S)	67.69 (S)	90.32 (F)
31	HSR21063-12	23.33 (N)	19.82 (N)	12.21 (N)	17.40 (N)	54.83 (S)	90.67 (F)	103.19 (F)
32	HSR21064-1	23.01 (N)	23.80 (N)	18.51 (N)	29.13 (N)	61.12 (S)	85.71 (F)	105.72 (F)
33	HSR21064-2	23.54 (N)	17.63 (N)	12.90 (N)	D	-	-	-
34	HSR21064-3	24.66 (N)	13.60 (N)	D	-	-	-	-
35	HSR21064-4	26.03 (N)	18.22 (N)	16.41 (N)	25.30 (N)	47.21 (S)	85.78 (F)	112.37 (F)
36	HSR21064-5	22.10 (N)	22.74 (N)	20.44 (N)	31.34 (N)	53.60 (S)	84.72 (F)	103.28 (F)
37	HSR21064-6	22.92 (N)	23.05 (N)	22.02 (N)	22.42 (N)	42.41 (S)	66.25 (S)	79.45 (F)
38	HSR21064-7	26.62 (N)	21.44 (N)	17.51 (N)	23.71 (N)	64.70 (S)	113.10 (F)	125.37 (F)
39	HSR21064-8	21.06 (N)	22.67 (N)	24.80 (N)	25.70 (N)	36.14 (N)	29.36 (N)	42.91 (S)
40	HSR21064-9	24.45 (N)	22.45 (N)	21.72 (N)	31.82 (N)	65.13 (S)	99.28 (F)	124.39 (F)
41	HSR21064-10	21.21 (N)	20.08 (N)	19.91 (N)	29.10 (N)	44.30 (S)	82.29 (F)	102.66 (F)
42	HSR21064-11	24.85 (N)	19.64 (N)	16.02 (N)	17.50 (N)	23.41 (N)	18.90 (N)	25.42 (N)
43	HSR21064-12	22.09 (N)	20.61 (N)	17.71 (N)	21.03 (N)	30.60 (N)	28.03 (N)	23.20 (N)
44	HSR21064-13	25.93 (N)	21.03 (N)	19.70 (N)	25.10 (N)	57.01 (S)	83.26 (F)	102.52 (F)
45	HSR21064-14	23.80 (N)	22.45 (N)	21.22 (N)	28.14 (N)	65.30 (S)	76.96 (F)	94.28 (F)
46	HSR21064-15	25.35 (N)	21.87 (N)	18.90 (N)	19.33 (N)	30.92 (N)	32.54 (N)	39.70 (S)
47	HSR21064-16	25.46 (N)	20.55 (N)	16.91 (N)	21.72 (N)	30.80 (N)	25.16 (N)	30.77 (N)
48	HSR21064-17	19.60 (N)	19.98 (N)	21.30 (N)	25.00 (N)	61.71 (S)	58.49 (S)	83.26 (F)
49	HSR21064-18	25.99 (N)	21.55 (N)	17.70 (N)	26.21 (N)	62.70 (S)	90.02 (F)	108.28 (F)
50	HSR21065-1	19.94 (N)	13.90 (N)	D	-	-	-	-
51	HSR21065-2	22.12 (N)	15.21 (N)	D	-	-	-	-
52	HSR21065-3	20.79 (N)	13.83 (N)	D	-	-	-	-
53	HSR21065-4	22.06 (N)	12.91 (N)	D	-	-	-	-
54	HSR21065-5	21.77 (N)	20.90 (N)	26.85 (N)	28.51 (N)	40.90 (S)	51.48 (S)	70.80 (S)
55	HSR21065-6	22.62 (N)	18.52 (N)	16.90 (N)	23.11 (N)	38.42 (S)	65.82 (S)	73.26 (S)
56	HSR21065-7	22.26 (N)	17.41 (N)	12.22 (N)	D	-	-	-
57	HSR21065-8	23.10 (N)	16.98 (N)	12.50 (N)	D	-	-	-
58	HSR21065-9	21.49 (N)	14.60 (N)	D	-	-	-	-
59	HSR21065-10	24.29 (N)	14.20 (N)	D	-	-	-	-
60	HSR21065-11	22.66 (N)	16.33 (N)	11.70 (N)	D	-	-	-
61	HSR21065-12	22.79 (N)	16.83 (N)	12.31 (N)	D	-	-	-
62	HSR21065-13	21.92 (N)	15.92 (N)	13.20 (N)	17.72 (N)	26.70 (N)	44.85 (S)	61.26 (S)
63	HSR21065-14	19.81 (N)	16.01 (N)	11.21 (N)	10.20 (N)	D	-	-
64	HSR21065-15	22.83 (N)	16.97 (N)	12.90 (N)	19.21 (N)	27.82 (N)	28.08 (N)	30.12 (N)
65	HSR21065-16	19.63 (N)	17.23 (N)	16.31 (N)	20.82 (N)	29.40 (N)	25.82 (N)	28.33 (N)
66	HSR21065-17	22.26 (N)	18.76 (N)	15.30 (N)	18.13 (N)	33.05 (N)	25.68 (N)	23.33 (N)

**Figure 2.**
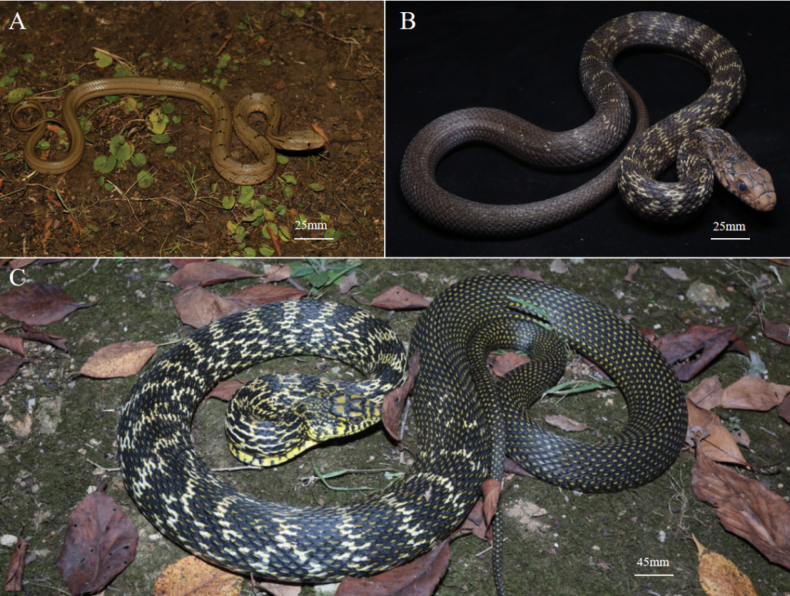
A. Neonatal-pattern of the King Ratsnake (HSR21061-7); B. Semi-pattern of the King Ratsnake (HSR21061-7); C. Full-pattern of the King Ratsnake (HSR21062).

We categorized the ontogenetic color pattern shift in *E. c. carinata* as three specific life stages described herein:

Neonatal-pattern (Fig. 2A): dorsum brown; two short black longitudinal stripes on neck; horizontal black stripes on front and middle dorsum (gradually disappearing on rear dorsum); four thin longitudinal black lines on dorsal tail; venter pink or yellowish-white.

**Figure 3.**
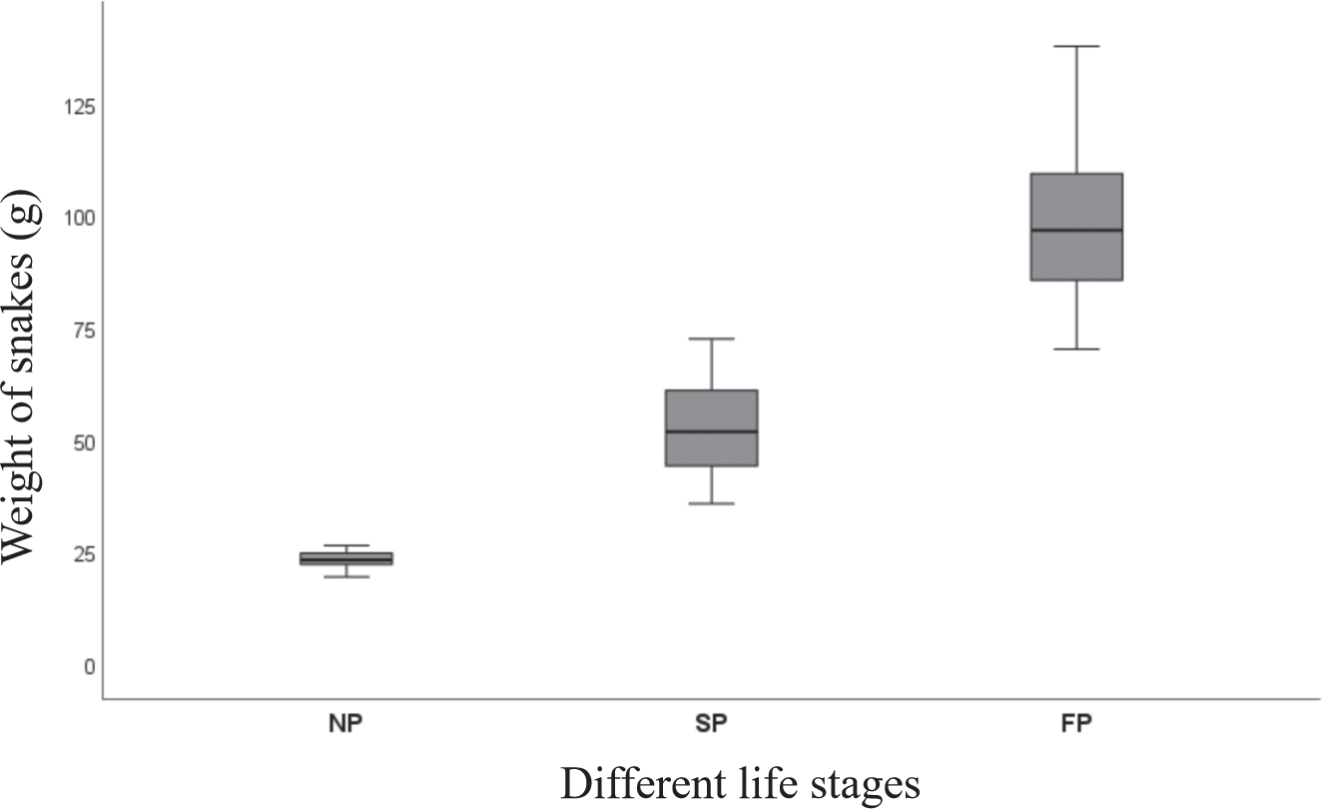
Results showing the three specific life stages and weights of *E. c. carinata*. The line inside the box indicates the median, the boxes are the first and third quartiles, and the whisker indicates the minimum and maximum variation.

Semi-pattern (Fig. 2B): ground color of dorsal body black; dorsal head dark brown and edges of each scale black; more than 20 irregular horizontal stripes on front and middle dorsum; rear body and tail are black with few spots. Center of the scales on dorsum lacking yellow spots (the main characteristic of the adult form).

Full-pattern (Fig. 2C): dorsal part of head, yellow and black edge of each scale appears to make up the Chinese two word of “big king”; the ground color of dorsal part of body, black with yellow spots present on center of most scales; more than 20 irregular horizontal light yellow stripes on front and middle dorsum (gradually disappear distally on dorsum); supralabials, infralabials and ventral part of the head mostly yellow; the edge of the ventral scales are black and form horizontal stripes, some individuals are completely black on rear venter.

We found that when the weight of the juvenile King Ratsnake reaches ~40 g, the neonatal-pattern gradually turns to the semi-pattern: the ground color of the dorsum gradually turns black; the black longitudinal lines on the neck disappeared; horizontal black stripes on front and middle dorsum changed from black to light yellow; and longitudinal lines on the rear lateral body and tail disappeared (Fig. 2B).

When the body weight is ~80 g, the semi-pattern gradually turns to the full-pattern: the center of each scale on the dorsum turns yellow (Fig. 1A).

When the experiment was completed on 22 June 2023, however, a subset of 15 individuals had consistently refused assisted feeding and had nearly stopped growing, and the color pattern of these individuals never changed.

## ﻿Discussion

Ontogenetic color pattern shift is common among snakes (Bechtel 1978; Burbrink 2001; Ernst and Ernst 2003). Some snakes’ color pattern shifts are complete, and neonatal snakes vary greatly in color pattern as adults, such as *E. c. carinata* (Zhao et al. 1998; Huang 2021). In other snakes, color pattern shifts are incomplete, such as *Achalinus meiguensis*, where the neonate has a white curved transverse spot about 2 mm wide on the occipital, which is absent in the adult. In this study, we subjectively divide ontogenetic color pattern shifts into complete ontogenetic color pattern shifts and incomplete ontogenetic color pattern shifts. However, at present, it is difficult to define the boundary between a complete ontogenetic color pattern shift and an incomplete ontogenetic color pattern shift, as the color pattern of the snake has not been quantified and is species-specific. Further research may be needed to quantify different color patterns for more accurate diagnosis of different degrees of ontogenetic color pattern shift.

Different color patterns are linked to a snakes’ extraordinary fitness (Madsen et al. 2006; Mochida et al. 2015; Goldenberg et al. 2024). For example, *Coluber constrictor* Linnaeus, 1758 has different antipredator behaviors between neonates and adults, utilizing different color patterns (Creer 2005). The understanding of ontogenetic color pattern shift in snakes is still insufficient. In the future, there is a lot of work to be done in investigating ecological value, molecular mechanisms, and evolutionary significance of ontogenetic color pattern shift.

In this study, we have shown that color pattern shift in the King Ratsnake, *Elaphe c. carinata*, is associated with body weight but not age. However, body length, number of sheds, and type of food were not considered (Stevens et al. 2014; Francis et al. 2023). Additional factors concerning ontogenetic color pattern shift should be considered in future research. We hope that the data in this study will be useful for the study of ontogenetic color pattern shifts in the future.

## ﻿Additional information

### Use of AI

No use of AI was reported.

### Author ORCIDs

Zhihao Jiang https://orcid.org/0009-0004-1492-0665

Yi Zhang https://orcid.org/0000-0002-0222-2366

Song Huang https://orcid.org/0000-0001-6786-8523

Ruyi Huang https://orcid.org/0000-0002-1004-3835

Tierui Zhang https://orcid.org/0009-0000-1869-9313

Yanan Gong https://orcid.org/0000-0002-8155-7024

Jinmin Chen https://orcid.org/0000-0001-6432-7721

### Data availability

All of the data that support the findings of this study are available in the main text.
